# Between-Subject and Within-Subject Variation of Muscle Atrophy and Bone Loss in Response to Experimental Bed Rest

**DOI:** 10.3389/fphys.2021.743876

**Published:** 2022-02-22

**Authors:** Jonas Böcker, Marie-Therese Schmitz, Uwe Mittag, Jens Jordan, Jörn Rittweger

**Affiliations:** ^1^Department of Muscle and Bone Metabolism, German Aerospace Center, Institute of Aerospace Medicine, Cologne, Germany; ^2^Institute of Medical Biometry, Informatics and Epidemiology (IMBIE), University Hospital Bonn, Bonn, Germany; ^3^Chair of Aerospace Medicine, University of Cologne, Cologne, Germany; ^4^German Aerospace Center, Head of Institute of Aerospace Medicine, Cologne, Germany; ^5^Department of Pediatrics and Adolescent Medicine, University Hospital of Cologne, Cologne, Germany

**Keywords:** between-subject variation, within-subject variation, measurement uncertainty, bed rest, muscle atrophy, bone loss

## Abstract

To improve quantification of individual responses to bed rest interventions, we analyzed peripheral quantitative computer tomography (pQCT) datasets of the lower leg of 76 participants, who took part in eight different bed rest studies. A newly developed statistical approach differentiated measurement uncertainty *U*_*Meas*_ from between-subject-variation (BSV) and within-subject variation (WSV). The results showed that *U*_*Meas*_ decreased 59.3% to 80% over the two decades of bed rest studies (*p* < 0.01), and that it was higher for muscles than for bones. The reduction of *U*_*Meas*_ could be explained by improved measurement procedures as well as a higher standardization. The majority (59.1%) of the individual responses *pc*_*i*_ exceeded the 95% confidence interval defined by *U*_*Meas*_, indicating significant and substantial BSV, which was greater for bones than for muscles, especially at the diaphyseal measurement sites. Non-significant to small positive inter-site correlations between bone sites, but very large positive inter-site correlation between muscle sites suggests that substantial WSV exists in the tibia bone, but much less so in the calf musculature. Furthermore, endocortical circumference, an indicator of the individual’s bone geometry could partly explain WSV and BSV. These results demonstrate the existence of substantial bone BSV, and that it is partly driven by WSV, and likely also by physical activity and dietary habits prior to bed rest. In addition, genetic and epigenetic variation could potentially explain BSV, but not WSV. As to the latter, differences of bone characteristics and the bone resorption process could offer an explanation for its existence. The study has also demonstrated the importance of duplicate baseline measurements. Finally, we provide here a rationale for worst case scenarios with partly effective countermeasures in long-term space missions.

## Introduction

Microgravity exposure is associated with profound adaptations of the human body, including muscle atrophy and bone loss, both of are usually interpreted as a result of unloading and the lack of mechanical forces (Vico et al., 2000; LeBlanc et al., 2007; Fitts et al., 2010; Rittweger et al., 2018). Especially, the lower limb is affected by these adaptations resulting in muscle wasting of 6 and 24% of the baseline volume after 8 days and 6 month of microgravity exposure, respectively (LeBlanc et al., 1997; Narici and de Boer, 2011). Bone losses amount to an average loss rate of 1–1.5% bone mineral content (BMC) per month (Pavy-Le Traon et al., 2007), which, however, is subject to substantial between-subject variation (BSV). Furthermore, besides BSV there is a within-subject variation (WSV) shown by differences in bone loss at different body sites after bed rest (Rittweger et al., 2005), the latter being a suitable analog of microgravity exposure on earth. The −6° head down tilt (HDT) bed rest induces an unloading of muscles and bones of the lower extremities and the spine evoking adaptations that are comparable to those from space-related adaptations. Bed rest studies unanimously show loss of muscle volume, of muscle strength and of BMC (Hargens and Vico, 2016). The loss of the muscle volume is exponential and in a comparable manner followed by losses in muscle power and muscle strength (Narici and de Boer, 2011). After 7–14 days of unloading, there are moderate effects, larger effects occur within 35 days of bed rest (Winnard et al., 2019). Furthermore, there is a correlation between reduction in muscle cross-sectional area (CSA) and strength loss. Because of that, CSA is a good approximation for muscle strength (Maughan et al., 1983; Rittweger et al., 2000; Fukunaga et al., 2001). Mitigating these profound adaptations and maintaining a sufficient physiological status is the aim of suitable countermeasures (Winnard et al., 2019). Yet, different training effort using such countermeasures can lead to variations in the musculoskeletal response (Timmons, 2011; Rittweger et al., 2018), however, in contrast, comparable training effort can also lead to BSV in muscle loss (English et al., 2015) or bone loss (Sibonga et al., 2015). The variation may be explained by the fact that there are responders and non-responders toward a training intervention (Mann et al., 2014; Hecksteden et al., 2015; Ahtiainen et al., 2016) resulting in a BSV (McPhee et al., 2010; Ross et al., 2019).

Until now, there are few approaches for quantifying BSV and WSV in response to bed rest (Narici and de Boer, 2011; Debevec et al., 2018; Scott et al., 2021). Thus, previous approaches have compared the standard deviation (SD) of changes between intervention and control group (Atkinson and Batterham, 2015; Hopkins, 2015). But so far, most researchers do not perform such analysis getting information about individual differences (Atkinson and Batterham, 2015). Moreover, systematic attempt to separate BSV and WSV from measurement-related uncertainty are rare except Swinton et al. (2018). Following Scott et al. (2021), bed rest is a good possibility to quantify “true” and “false” individual differences, because due to the conditions of such a study, several influencing parameters like nutrition or daily activities are controlled. Atkinson et al. (2019) explained that there is an additional factor, which cannot be controlled. There is a random WSV due to the period of time between the baseline data collection (BDC) and the follow up measurements, mostly influenced by non-standardized behavior of the participants. Understanding BSV and WSV is of general interest. Thus, large BSV in the presence of small WSV would favor genetic and epigenetic mechanisms, whilst large BSV in combination with large WSV would require more complicated explanation models. Moreover, detailed information about BSV and WSV is highly relevant in practical terms when the health and well-being of single individuals is at stake. Therefore, the aim of the present work was to explore variation in musculoskeletal responses to bed rest, and to separate BSV, WSV and measurement-related uncertainty.

## Materials and Methods

### Selected Studies

We selected data sets of eight bed rest studies (AGBRESA, BBR, LTBR, MEP, NUC, Planhab, RSL, Valdoltra), which took place in France, Slovenia, and Germany, respectively, from 2001 till 2019 to explore the variation in musculoskeletal responses after bed rest. The studies varied in bed rest duration as well as bed rest condition (horizontal bed rest or head down tilt bed rest). The full study names, the year, the location, and the characteristics, which were important for this manuscript, are presented in Table 1. Additionally, Table 1 includes references, which explain the exact study details.

**TABLE 1:** Overview about the included studies with information about the year, the location, the number of subjects, the duration of bed rest, bed rest position, and measurement sites.

k	Study	Full study name	Year	Location	Number of subjects (*n*_*k*_)	Duration of bed rest	Position	Muscle sites	Bone sites	References
1	AGBRESA	Artificial Gravity Bed Rest with ESA	2019	DLR, Cologne (GER)	8	60	HDT	TIBIA_38, TIBIA_66	TIBIA_04, TIBIA_38, TIBIA_66, TIBIA_98	Frett et al., 2020
2	BBR	Berlin Bed Rest	2003/2004	Benjamin Franklin Campus, Berlin (GER)	10	56	HDT	TIBIA_66	TIBIA_04, TIBIA_38, TIBIA_66	Rittweger et al., 2006
3	LTBR	Long-Term Bed Rest	2001/2002	MEDES, Toulouse (FR)	9	90	HDT	TIBIA_66	TIBIA_04, TIBIA_66	Rittweger et al., 2005
4	MEP	Medium-term Bed Rest Whey Protein Study	2011/2012	DLR, Cologne (GER)	8	21	HDT	-	TIBIA_04, TIBIA_38, TIBIA_66	Blottner et al., 2014
5	NUC	Nutritional Countermeasure	2010	DLR, Cologne (GER)	7	21	HDT	-	TIBIA_04, TIBIA_38, TIBIA_66	Heer et al., 2014
6	Planhab	Planetary Habitat Simulation Study	2012/2013	Olympic Sports Center, Planica (SLO)	13	21	HBR	TIBIA_66	TIBIA_04, TIBIA_38, TIBIA_66, TIBIA_98	Debevec et al., 2014
7	RSL	Reactive Jumps in a Sledge Jump system as a countermeasure during long-term bed rest	2015/2016	DLR, Cologne (GER)	11	60	HDT	TIBIA_38, TIBIA_66	TIBIA_04, TIBIA_38, TIBIA_66, TIBIA_98	Kramer et al., 2017
8	Valdoltra	Valdoltra	2007	Orthopedic Hospital, Valdoltra (SLO)	10	35	HBR	-	TIBIA_04, TIBIA_38, TIBIA_98	Borina et al., 2010

*HDT, 6° head down tilt bed rest; HBR, horizontal bed rest.*

*The position with 6° head down tilt (HDT) and horizontal bed rest (HBR), the observed measurement sites for the muscles and the bones.*

Three out of the eight studies (LTBR, RSL, and Valdoltra) included two baseline measurements of the peripheral quantitative computed tomography (explained later on). This enabled a calculation of the measurement uncertainty, because the time between the two measurements was short and no intervention took place. As it is very consistent in literature, the bone losses, but not muscle wasting, continue for another 10–20 days following re-ambulation (Rittweger et al., 2005, 2010; Hargens and Vico, 2016), muscle losses were quantified at the last day of bedrest and bone losses at 14 days of follow-up after re-ambulation. Due to comparable durations of bed rest, BBR was presented as AGBRESA and RSL with 60 days of bed rest in the results section.

### Participants

In total, we analyzed datasets of 76 participants, who took part in the described eight different bed rest studies. For the purpose of this manuscript only the data of the control groups, which experienced no intervention excluding bed rest, were analyzed. Only for calculation of the measurement uncertainty of LTBR, RSL and Valdoltra we used the data sets of control and intervention group getting more precise values. Additionally, from 11 subjects of the LTBR study (control and intervention group), both legs were measured and were also used for analysis of the measurement uncertainty.

### Peripheral Quantitative Computed Tomography Measurements

**FIGURE 1:**
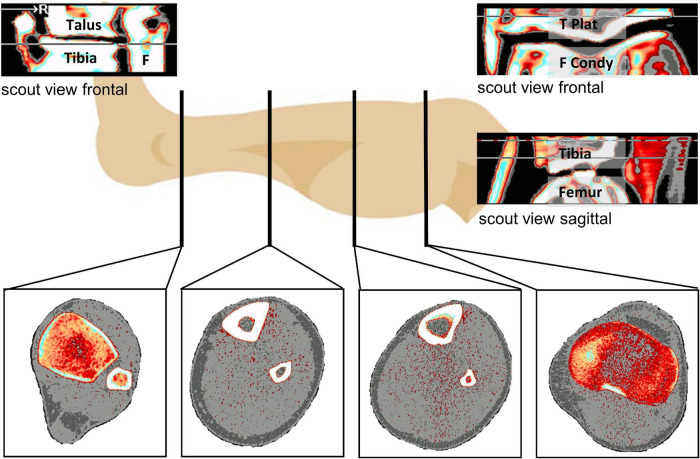
Overview about the analyzed measurement sites (at the bottom) from left to right with TIBIA_04 (Epiphysis at the ankle), TIBIA_38 and TIBIA_66 at the diaphysis and TIBIA_98 (Epiphysis at the knee joint). 04, 38, 66, and 98 represent the relative position regarding the tibia length from ankle joint to knee joint. Top left is the distal frontal scout view (Tibia, Talus, F: Fibula), top right the frontal proximal scout view (F Condy: Femur Condyles, T Plat: Tibia Plateau), below that the sagittal proximal scout view.

The tibia is composed of (1) distal and proximal epiphysis, which are both rich in trabecular bone and devoid of thick compact bone, (2) distal and proximal metaphysis, which are composed both of compact and trabecular bone, and (3) the diaphysis (shaft), which is mainly composed of thick compact bone and almost devoid of trabecular bone (Capozza et al., 2010), whereas at the proximal epiphysis the cortical shell consisting of compact bone is often not thicker than trabeculi. At diaphyseal sites, pQCT also allows segmentation of muscle from skin and bone tissues, thereby yielding the anatomical muscle cross-sectional area (CSA), albeit without distinction of individual muscle groups (Figure 1). All selected studies included a pQCT measurement with either a XCT2000 or a XCT3000 device (Stratec Medizintechnik, Pforzheim, Germany) (Table 2). The measurement process for pQCT has been described in detail elsewhere (Rittweger et al., 2010). In brief, the XCT devices use single-beam rotation-translation CT technology, and distal and proximal tibial sites are identified from tibio-talar and femoro-tibial scout views, respectively, whilst sites at the diaphysis are either found manually or *via* a scout view (Figure 1). Over the two decades that the studies have been performed, the procedures have been slightly modified to maximize repeatability of the measurements. Moreover, whilst earlier studies had solely focused on the distal tibia, the proximal tibia has also been studied since 2007, initially using femoro-tibial scout views in the frontal plane, and with sagittal scout views since 2016 (Table 2). As bed rest induces minimal to no changes in the metaphysis and in the forearms, the present manuscript focusses on epiphyseal and diaphyseal bone sites including two muscles sites at the diaphysis, thereby allowing WSV assessment in addition to BSV.

**TABLE 2:** Overview about the way of the determination of the measurement site of TIBIA_98 and TIBIA_66.

k	Study	pQCT device	Scout view orientation at TIBIA_98	TIBIA_66 detection
1	AGBRESA	XCT3000	Sagittal	Automatic
2	BBR	XCT2000	Frontal	Manual
3	LTBR	XCT2000	Frontal	Manual
4	MEP	XCT3000	Frontal	Manual
5	NUC	XCT3000	Frontal	Manual
6	Planhab	XCT3000	Frontal	Automatic
7	RSL	XCT3000	Sagittal	Automatic
8	Valdoltra	XCT2000	Frontal	Automatic

### Image Analysis and Data Processing

All image analysis was performed with the integrated XCT software Stratec XCT Console in its version 6.20 in accordance with the manufacturer’s instructions. The operator marked the regions of interest (ROI) in each picture (tibia, fibula, muscle including tibia and fibula at 38 and 66% of tibia length) and defined the segmentation threshold for the outer bone or muscle contour. The threshold differed between measurement sites, studies, and could even differ between subjects, respectively. The threshold was chosen yielding a satisfactory contour of the ROI and was kept for each subject throughout the study. To differentiate between compact and trabecular bone tissue at the epiphyseal measurement sites, we run a second analysis with a segmentation threshold at 650 mg/cmł, which enabled quantifying the compact bone at these sites (Rittweger et al., 2010). After analyzing all pQCT scans, an analysis was performed by the XCT software that encompassed all CT numbers included in the present analysis. The resulting data base was further processed with custom-made R-scripts (Version 3.6.0^1^), using the RStudio environment (Version 1.2.1335, Boston, MA, United States). Thus, BMC was identified as the product of the XCT-variables “TOT_DEN” and “TOT_A” divided by 1,000. The endocortical circumference (ENDO) was identified from “ENDO_C”. Muscle CSA was calculated as the differences between “TOT_A” of the ROI of the muscle and “TOT_A” of tibia and fibula.

### Statistical Computation

We assumed that measurement uncertainty (*U*_*Meas*_) was variable across measurement sites and studies, but invariant within a given study and measurement site. Moreover, we assumed that *U*_*Meas*_ and the individual response to the bed rest intervention (*U*_*IR*_) depict normal distribution. It follows then from the principle of linear superposition that the observed response’s uncertainty *U*_*Obs*_ can be conceptualized as the sum of these two parameters.


(1) $$U_{O\,b\,s}=U_{M\,e\,a\,s}+U_{I\,R}$$

We defined the observed individual loss of bone or muscle of subject i (i = 1, …, *n*_*k*_ with *n*_*k*_ as the total number of subjects of study *k, k = 1, …, 8*) after bed rest intervention as the percent change *pc*_*i*_:


(2) $$p\,c_{i\,k}=\frac{x_{I\,R,i\,k}-x_{B\,D\,C,i\,k}}{x_{B\,D\,C,i\,k}}\times 100$$

*pc*_*ik*_ is defined as the difference either BMC or CSA between the individual result after the intervention (*x_*IR*,ik_*) and the result of the first baseline measurement (*x*_*BDC,ik*_) divided by the baseline result (*x*_*BDC,ik*_). This result is multiplied by 100 getting the percent change *pc*_*ik*_. The mean among all subjects of a study is denoted by *pc*_*k*_ and is calculated for each measurement site of each of the eight included studies.

Empirically, the observed uncertainty $\hat{U}$*_*Obs*,k_* (*k* indicating the study) could be assessed as the variance of the individual percent change *pc*_*ik*_ of each participant of each study’s control group, *n*_*k*_ is indicating the number of participants in the control group of study *k*, and $\bar{p\,c_{k}}$ the mean percent change of these participants:


(3) $${\hat{U}}_{O\,b\,s,k}=\frac{1}{n_{k}-1}\,\sum_{i=1}^{n_{k}}{\left(p\,c_{i\,k}-\bar{p\,c_{k}}\right)}^{2}$$

$\hat{U}$*_*Meas*_* denote measurement uncertainties, which are usually assessed during baseline through repetition of measurements. Typically, we use two baseline measurements (*n*_*i*_ = 2) to compute the variance within each participant. The mean of these variances of all participants of one study and measurement site represents ${\bar{U}}_{M\,e\,a\,s,k}$. As we compared individual’s response toward the measurement uncertainty, the calculation of the variance was more practical resulting in absolute values compared to the coefficient of variation. For calculation of ${\bar{U}}_{M\,e\,a\,s,k}$ we used the baseline measurements of all subjects of study *k*, irrespective of the group (control or intervention group) to get more precise values. *n*_*i*_ represents the number of baseline measurements, *x*_*BDC,mk*_ the value of each baseline measurement (BMC or CSA), and $\bar{x_{B\,D\,C,k}}$ the mean of all baseline measurements of the specific participant.


(4a) $${\hat{U}}_{M\,e\,a\,s,i\,k}=\left(\frac{1}{n_{i}-1}\,\sum_{m=1}^{n_{i}}{\left(x_{B\,D\,C,m\,k}-\bar{x_{B\,D\,C,k}}\right)}^{2}\right)\times {\left(\frac{100}{\bar{x_{B\,D\,C,k}}}\right)}^{2}$$

(4b) $${\bar{U}}_{M\,e\,a\,s,k}=\frac{\sum_{i=1}^{n_{k}}{\hat{U}}_{M\,e\,a\,s,i\,k}}{n_{k}}$$

Solving Equation 1 for $\hat{U}$*_*IR*_* and replacing the parameters yields


(5) $${\hat{U}}_{I\,R,k}={\hat{U}}_{O\,b\,s,k}-{\bar{U}}_{M\,e\,a\,s,k}$$

$${\hat{U}}_{I\,R,k}=\frac{1}{n_{k}-1}\,\sum_{i=1}^{n_{k}}{\left(p\,c_{i\,k}-\bar{p\,c_{k}}\right)}^{2}-\frac{\sum_{i=1}^{n_{k}}{\hat{U}}_{M\,e\,a\,s,i\,k}}{n_{k}}$$

This variable has been obtained for each study and measurement site. As only LTBR, RSL and Valdoltra included two baseline measurements, it was impossible to calculate ${\bar{U}}_{M\,e\,a\,s,k}$ for all study and measurement sites. So, for studies with only one baseline measurement, we used the result of the study with the most similar measurement condition (Table 2). If this was not possible due to missing data (e.g., Valdoltra provided no data about MUSCLE_66, which is needed for Planhab MUSCLE_66 calculations), we used the higher ${\bar{U}}_{M\,e\,a\,s,k}$ of the other studies (in the case of the example, we used ${\bar{U}}_{M\,e\,a\,s,k}$ of LTBR).

Additionally, to compare results of studies with different durations of bed rest, we introduced the adjusted between subject deviation (ABD) for each study *k*. *ABD*_*k*_ is defined as the square root of the uncertainty of individual response divided by the mean of the percent change of each study and measurement site. This result is divided by each study’s duration in weeks. By using the mean percent change and the study duration, we normalized the uncertainty to bed rest duration and bed rest conditions as well as other factors like age or sex of the participants:


(6) $$A\,B\,D_{k}=\frac{\sqrt{{\hat{U}}_{I\,R,k}}}{{\bar{p\,c}}_{k}}÷w\,e\,e\,k\,s$$

Besides these analyses of variances, we calculated the absolute bone loss (BL) as difference of BMC of the first baseline measurement and the post bed rest value:


(7) $$B\,L_{i\,k}=B\,M\,C_{B\,D\,C,i\,k}-B\,M\,C_{I\,R,i\,k}$$

### Between-Subject Variation and Within-Subject Variation

The main focus of this manuscript is on the between-subject variation (BSV) and the within-subject variation (WSV) separated from the measurement uncertainty. For computation of the BSV, we calculated the mean percent change of each measurement site and study. Furthermore, we defined a 95%-confidence interval by using the term 1.96 ⋅ *U*_*Meas*_ (measurement uncertainty of the specific measurement site and study or the most similar condition as described earlier). Thus, the interval was defined as the range between mean percent change added up and subtracted by the term based on the measurement uncertainty. If the individual percent change of a participant exceeded this interval, there was a BSV.

We defined WSV in this manuscript that there was no correlation between loss of muscle volume and/or BMC within one subject along the measurement sites. For this purpose, we used the Pearson’s correlation coefficient between the different measurement sites for each study. As there are differences regarding the bone tissue content between the bone sites, we additionally analyzed the loss of compact and trabecular bone loss and its relationships toward the other measurement sites.

### Statistics

All computations and statistics were performed with RStudio (Version 1.2.1335, Boston, MA, United States) based on the R-environment (Version 3.6.0,see text footnote 1). Firstly, a mean value of the baseline BMC of the studies with two baseline measurements was calculated followed by calculation of the difference from the mean to the first baseline data collection (BDC1) and was checked for normal distribution. To assess changes of BMC and CSA after the bed rest intervention to BDC1, a paired sample *t*-test was performed separately for each measurement site and study. An ANOVA was used to evaluate differences in *U*_*Meas*_ (studies with two baseline measurements: LTBR, RSL, Valdoltra). The mean of *U*_*Obs*_ and *U*_*IR*_ for each measurement site was compared by an ANOVA. In case of a significant result for the *F*-test of the ANOVA, a *post-hoc* Tukey Honest Significant Differences test was performed to further analyze differences between the studies and measurement. BSV is shown by exceeding the 95%-confidence interval, which was calculated based on *U*_*Meas*_ (as described before). The correlations of the percent changes for muscles and bones were analyzed by the Pearson correlation coefficient and scored as defined by Hinkle et al. (2009). Based on these results of the Pearson’s correlation coefficient we could do statements about WSV. The relationship between ENDO and BL was analyzed by a linear regression analysis for each study and all studies combined. This approach was performed based on the results of Rittweger et al. (2009) and adapted that we did not use the compact bone loss, but the total BMC loss. Finally, we fitted a linear mixed model (LMM) for analyzing the outcome variables BMC and CSA with fixed effects of measurement site, study days, amount of bed rest and ENDO (only for BMC), respectively, and random intercepts for study and subjects within a study allowing different variances for each measurement site (in case of heteroscedasticity of the within-group errors).

## Results

**FIGURE 2:**
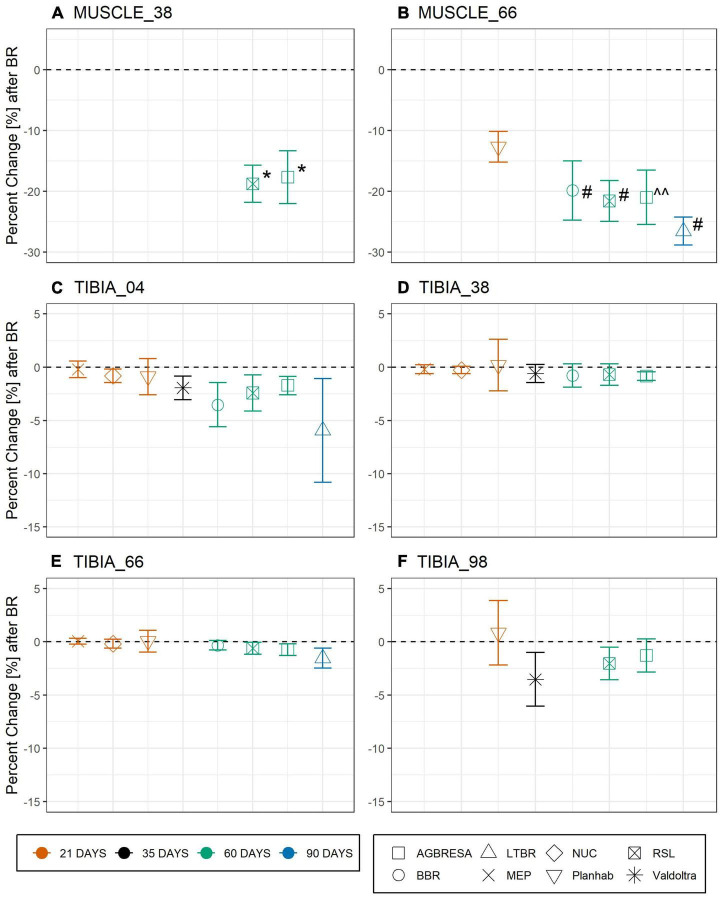
Percent change pc_*k*_ as response toward bed rest intervention on the basis of muscle cross section area (CSA) for **(A)** MUSCLE_38 (RSL: *p* = 0.0018; AGBRESA *p* = 0.0033) and **(B)** MUSCLE_66 (BBR: *p* = 6.58-04; RSL: *p* = 0.0002; AGBRESA: *p* < 0.05; LTBR: *p* = 3.35–07) and bone mineral content (BMC) for **(C)** TIBIA_04, **(D)** TIBIA_38, **(E)** TIBIA_66, and **(F)** TIBIA_98, respectively. Numbers indicate the relative measurement position regarding the entire tibia length from distal to proximal. The average response is shown, error bars represent standard deviation. The color indicates the bed rest duration and the shape represents the study. ^^ denotes significant changes with *p* < 0.05; * denotes significant changes with *p* < 0.01; ^#^ denotes significant changes with *p* < 0.001.

Differences between values of baseline data collection 1 (BDC1) and the within-subject means of the two baseline measurements were near-normally distributed without any substantial deviation, thereby justifying the approach outlined in the equations above. The difference between BDC1 and post bed rest was significant for MUSCLE_38 (AGBRESA: *p* = 0.003 and RSL: *p* = 0.002) and MUSCLE_66 for AGBRESA (*p* = 0.01), BBR (*p* < 0.001), LTBR (*p* < 0.001) and RSL (*p* < 0.001), but not for the 21-day Planhab study (*p* = 0.07) (Supplementary Table 1). Figure 2 shows the study’s percent change at each measurement site.

**FIGURE 3:**
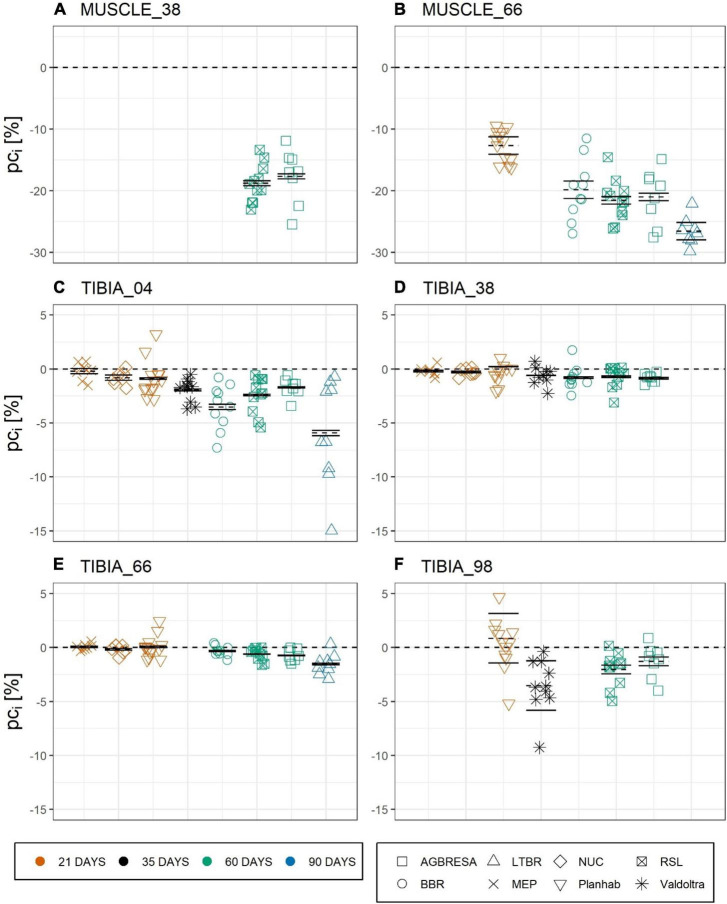
Chart of the individual percent change (pc_*i*_) by measurement sites with **(A)** CSA at MUSCLE_38, **(B)** CSA at MUSCLE_66, **(C)** BMC at TIBIA_04, **(D)** BMC at TIBIA_38, **(E)** BMC at TIBIA_66, and **(F)** BMC at TIBIA_98, where the numbers indicate the relative measurement position regarding the entire tibia length from distal to proximal. The color indicates the bed rest duration and the shape represents the study. Each chart is separated by the studies that performed measurements at the measurement site. Mean of the pc as dashed line, upper and lower limit of the 95%-confidence interval based on measurement uncertainty U_*Meas*_ as solid lines. Most pc*i* exceed the confidence interval, indicating significant between-subject variation.

For computation of the measurement uncertainty *U*_*Meas*_, we used the results of the studies with two baseline measurements, meaning LTBR, RSL, and Valdoltra, respectively. ANOVA indicated a significant difference of *U*_*Meas*_ between RSL and LTBR at TIBIA_66 (*p* = 0.005) (Table 3). As can be seen from Figure 3, the majority (59.1%) of the observed individual percent change *pc*_*i*_ exceeds the confidence intervals, indicating significant and substantial BSV. However, the BSV was greater for bone (65.9%) than for muscle (34.8%), and the BSV was greater for the diaphysis (71.0%) than for the epiphyseal measurement sites (60.2%). By subtracting the calculated *U*_*Meas*_ from *U*_*Obs*_, *U*_*IR*_ was calculated (Table 4).

**TABLE 3:** Measurement Uncertainty U_*Meas*_ [%^2^] of studies with two baseline measurements per body site.

	LTBR	Valdoltra	RSL	Mean	*p*-value
MUSCLE_38	-	-	0.73	0.73 ± 1.02	-
MUSCLE_66	2.95	-	1.20	2.26 ± 3.50	0.053
TIBIA_04	0.38	0.08	0.09	0.24 ± 0.56	0.10
TIBIA_38	-	0.02	0.10	0.09 ± 0.16	0.16
TIBIA_66	0.15	-	0.03	0.10 ± 0.16	0.005
TIBIA_98	-	1.92	0.60	1.00 ± 2.22	0.12

*Mean and standard deviation (SD) are shown. Mean and SD were computed by including all values of all studies, which provided data for the specific measurement site. P-value represents the results of ANOVA for analyzing significant differences for U_Meas_.*

**TABLE 4:** Overview about Observed Uncertainty U_*Obs*_ [%^2^] and Uncertainty of individual response U_*IR*_ [%^2^] by studies and body sites.

	AGBRESA		BBR		LTBR		MEP		NUC		Planhab		RSL		Valdoltra	
	*U*_*Obs*_	*U*_*IR*_	*U*_*Obs*_	*U*_*IR*_	*U*_*Obs*_	*U*_*IR*_	*U*_*Obs*_	*U*_*IR*_	*U*_*Obs*_	*U*_*IR*_	*U*_*Obs*_	*U*_*IR*_	*U*_*Obs*_	*U*_*IR*_	*U*_*Obs*_	*U*_*IR*_
MUSCLE_38	18.85	18.12	-	-	-	-	-	-	-	-	-	-	9.35	8.62	-	-
MUSCLE_66	19.96	18.77	23.56	20.63	5.37	2.42	-	-	-	-	6.36	3.41	11.30	10.10	-	-
TIBIA_04	0.73	0.63	4.27	3.89	23.62	23.25	0.61	0.24	0.40	0.02	2.81	2.74	2.81	2.72	1.22	1.15
TIBIA_38	0.17	0.06	1.20	1.10	-	-	0.18	0.08	0.12	0.02	5.88	5.86	1.01	0.91	0.73	0.72
TIBIA_66	0.31	0.28	0.21	0.06	0.89	0.75	0.08	-0.07*	0.18	0.04	1.04	0.89	0.32	0.29	-	-
TIBIA_98	2.39	1.79	-	-	-	-	-	-	-	-	9.20	7.28	2.32	1.72	6.37	4.44

*Negative value, because there was no bone loss during MEP at TIBIA_66 and U_Meas_ was subtracted.

*U*_*Obs*_ and *U*_*IR*_ were significantly greater for MUSCLE_66 compared to TIBIA_38 (*p* = 0.009 and *p* = 0.04) and TIBIA_66 (*p* = 0.005 and *p* = 0.02), as well as for MUSCLE_38 compared to TIBIA_66 (*p* = 0.03) (Supplementary Table 2). Generally, it could be seen a trend that both *U*_*Obs*_ and *U*_*IR*_ were greater for the muscle measurement sites than for the bone measurement sites, except for LTBR, where *U*_*Obs*_ and *U*_*IR*_ were greater for TIBIA_04 than for MUSCLE_66.

**FIGURE 4:**
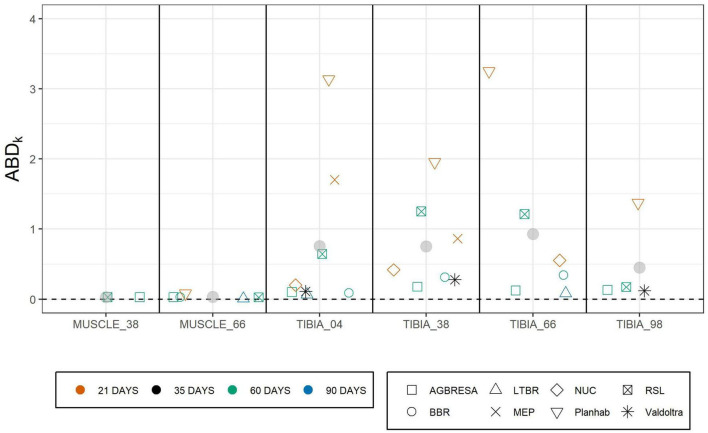
Adjusted between subject deviation (ABD_*k*_) of all studies separated by measurement sites. ABD_*k*_ represented the square root of uncertainty of individual response U_*IR*_ divided by pc_*k*_ enabling the comparison of several studies with different bed rest durations. The color indicates the bed rest duration and the shape represents the study. The data of MEP for TIBIA_66 are missing due to negative values of the uncertainty of individual response U_*IR*_. Gray points represent the mean value of the specific measurement site.

**FIGURE 5:**
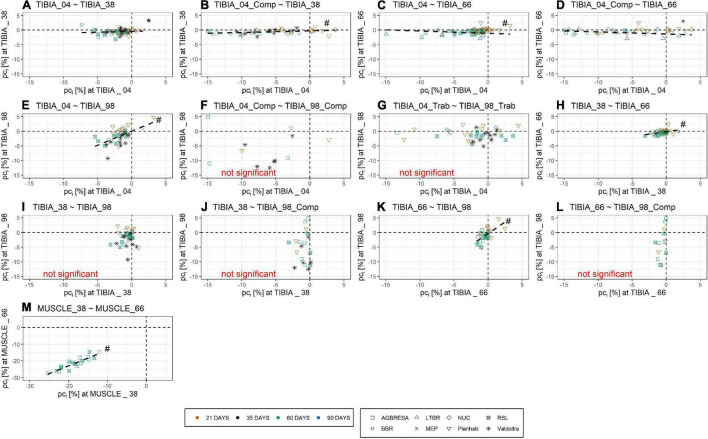
Pearson Correlation of pc_*i*_ of **(A)** TIBIA_04 and TIBIA_38, **(B)** TIBIA_04_Comp and TIBIA_38, **(C)** TIBIA_04 and TIBIA_66, **(D)** TIBIA_04_Comp and TIBIA_66, **(E)** TIBIA_04 and TIBIA_98, **(F)** TIBIA_04_Comp and TIBIA_98_Comp, **(G)** TIBIA_04_Trab and TIBIA_98_Trab, **(H)** TIBIA_38 and TIBIA_66, **(I)** TIBIA_38 and TIBIA_98, **(J)** TIBIA_38 and TIBIA_98_Comp, **(K)** TIBIA_66 and TIBIA_98, **(L)** TIBIA_66 and TIBIA_98_Comp, and **(M)** MUSCLE_38 and MUSCLE_66. Numbers indicate the relative measurement position regarding the entire tibia length from distal to proximal. Comp and Trab indicate compact and trabecular loss, respectively. The color indicates the bed rest duration and the shape represents the study. Several outliers are not shown in the figure due to graphical reasons, but are included in the linear regression analysis. Within the muscle sites, there was a strong positive correlation. The inter-site correlation of the bone sites ranged from no correlation to low positive correlation and moderate positive correlation. Strong positive correlation indicates no within-subject variation for the muscles, no correlation or low correlation indicates within-subject variation. Dashed line represents significant correlation. * denotes significant correlation with *p* < 0.01; ^#^ denotes significant correlation with *p* < 0.001.

As shown in Figure 4, the adjusted between-subject deviation *ABD*_*k*_ for the Planhab study depicts three outliers, namely for TIBIA_04 and TIBIA_66. In the absence of any bone loss for TIBIA_66 in the MEP study, *ABD*_4_ could not be computed. Figure 5 shows the results of the inter-site correlation analyses. A very high positive correlation of *pc*_*i*_ between MUSCLE_38 and MUSCLE_66 was observed (*r* = 0.90, *p* < 0.001). For BMC, there was no correlation seen between TIBIA_38 and TIBIA_98 (*r* = 0.29, *p* = 0.07), whereas the correlation ranged from 0.34 (TIBIA_04 and TIBIA_38; *p* = 0.006; low positive correlation) to 0.52 (TIBIA_38 and TIBIA_66; *p* < 0.001; moderate positive correlation) between the remaining bone site pairs. When differentiating compact and trabecular bone tissue at the epiphyseal bone sites, there were significant correlations between TIBIA_04_Comp and TIBIA_38 (*r* = 0.51; *p* < 0.001), and TIBIA_66 (*r* = 0.39; *p* = 0.002), respectively, but no correlation to TIBIA_98_Comp (*r* = 0.29; *p* = 0.19). Additionally, TIBIA_98_Comp showed no correlation to either TIBIA_38 (*r* = 0.12, *p* = 0.54) or TIBIA_66 (*r* = 0.10, *p* = 0.62). The loss of trabecular bone within the epiphyseal sites showed no correlation (*r* = −0.12, *p* = 0.36). With regards to muscle-bone inter-relationships, there was a moderate positive correlation between MUSCLE_38 and TIBIA_98 (*r* = 0.68, *p* = 0.001), MUSCLE_66 and TIBIA_66 (*r* = 0.56, *p* < 0.001) and MUSCLE_66 and TIBIA_98 (*r* = 0.56, *p* < 0.001), and a low positive correlation between MUSCLE_66 and TIBIA_04 (*r* = 0.47, *p* < 0.001) and MUSCLE_66 and TIBIA_38 (*r* = 0.34, *p* = 0.03; Table 5).

**TABLE 5:** Correlation (*p*-value and *r*) of percent change pc_*k*_ for CSA and BMC.

Measurement site	MUSCLE_38	MUSCLE_66	TIBIA_04_total	TIBIA_04_comp	TIBIA_04_trab	TIBIA_38	TIBIA_66	TIBIA_98_total	TIBIA_98_comp	TIBIA_98_trab
MUSCLE_38	-	*p* < 0.001 *r* = 0.90	*p* = 0.30 *r* = 0.25	-	-	*p* = 0.09 *r* = 0.40	*p* = 0.49 *r* = 0.17	*p* = 0.001 *r* = 0.68	-	-
MUSCLE_66	*p* < 0:001 *r* = 0.90	-	*p* < 0.01 *r* = 0.47	-	-	*p* = 0.03 *r* = 0.34	*p* < 0.001 *r* = 0.56	*p* < 0.001 *r* = 0.56	-	-
TIBIA_04_total	*p* = 0.30 *r* = 0.25	*p* < 0.01 *r* = 0.47	-	-	-	*p* = 0.006 *r* = 0.34	*p* < 0.001 *r* = 0.47	*p* < 0.001 *r* = 0.51	-	-
TIBIA_04_comp	-	-	-	-	-	*p* < 0.001 *r* = 0.51	*p* = 0.002 r = 0.39	-	*p* = 0.19 r = 0.29	-
TIBIA_04_trab	-	-	-	-	-	-	-	-	-	*p* = 0.36 *r* = −0.18
TIBIA_38	*p* = 0.09 *r* = 0.40	*p* = 0.03 *r* = 0.34	*p* = 0.006 *r* = 0.34	*p* < 0.001 *r* = 0.51	-	-	*p* < 0.001 *r* = 0.52	*p* = 0.07 *r* = 0.29	*p* = 0.54 *r* = 0.12	-
TIBIA_66	*p* = 0.49 *r* = 0.17	*p* < 0.001 *r* = 0.56	*p* < 0.001 *r* = 0.47	*p* = 0.002 *r* = 0.39	-	*p* < 0.001 *r* = 0.52	-	*p* < 0.001 *r* = 0.51	*p* = 0.62 *r* = 0.10	-
TIBIA_98_total	*p* = 0.001 *r* = 0.68	*p* < 0:001 *r* = 0.56	*p* < 0.001 *r* = 0.51	-	-	*p* = 0.07 *r* = 0.29	*p* < 0.001 *r* = 0.51	-	-	-
TIBIA_98_comp	-	-	-	*p* = 0.19 *r* = 0.29	-	*p* = 0.54 *r* = 0.12	*p* = 0.62 *r* = 0.10	-	-	-
TIBIA_98_trab	-	-	-	-	*p* = 0.36 *r* = −0.18	-	-	-	-	-

*Included were all datasets which provide data of both measurement sites. The epiphyseal measurement sites TIBIA_04 and TIBIA_98 were shown as total BMC and divided into compact (comp) and trabecular (trab) BMC. Significant correlations are marked in gray.*

**FIGURE 6:**
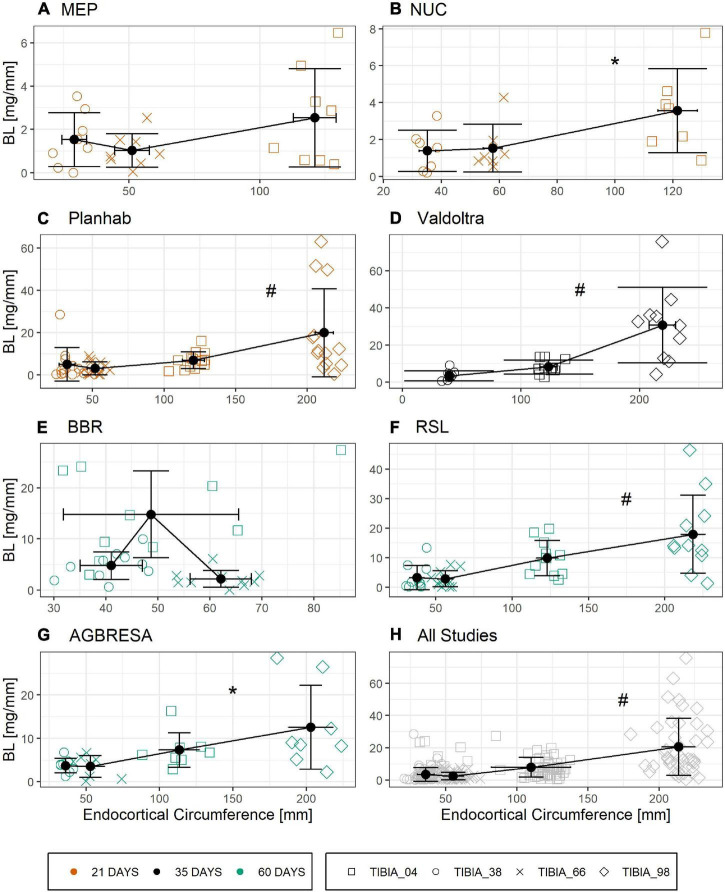
Linear relationship of Bone Loss BL in [mg/mm] and endocortical circumference ENDO [mm] divided by study with **(A)** MEP, **(B)** NUC, **(C)** Planhab, **(D)** Valdoltra, **(E)** BBR, **(F)** RSL, **(G)** AGBRESA, and (H) all studies. The color indicates the bed rest duration and the shape represents the measurement site. Numbers in the measurement site names indicate the relative measurement position regarding the entire tibia length from distal to proximal. Presented are studies with at least three out of four bone sites. Black points are the mean value of each measurement site with error bars of BL and ENDO. Linear regression analysis showed significant associations across the different studies except for BBR and MEP. * denotes significant relationship with *p* < 0.01; ^#^ denotes significant relationship with *p* < 0.001.

**FIGURE 7:**
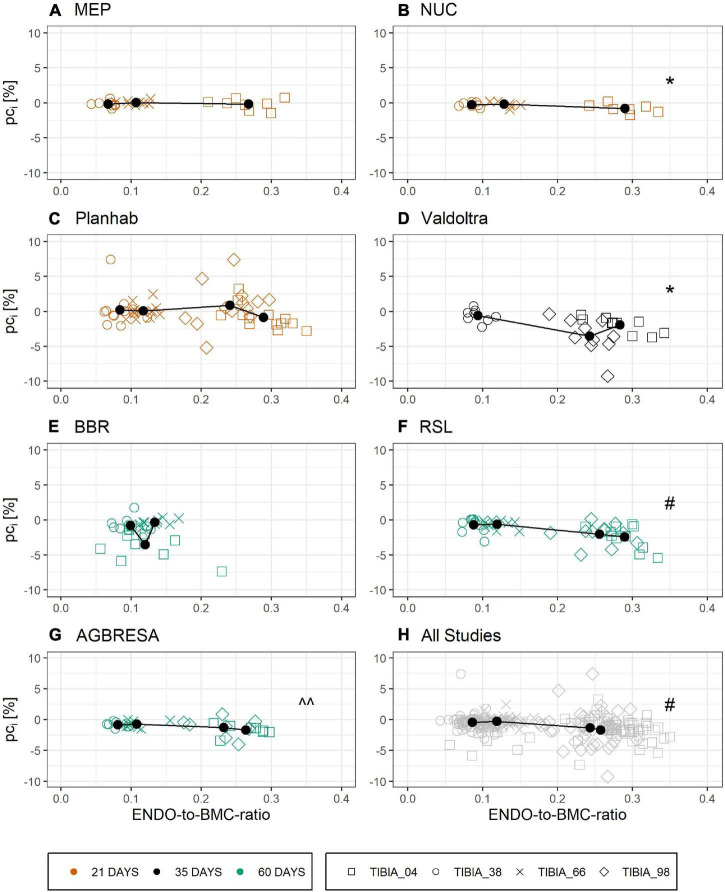
Linear relationship of ENDO-to-BMC-ratio at baseline and the individual pc_*i*_ by study with **(A)** MEP, **(B)** NUC, **(C)** Planhab, **(D)** Valdoltra, **(E)** BBR, **(F)** RSL, **(G)** AGBRESA, and **(H)** all studies. The color indicates the bed rest duration and the shape represents the measurement site. Numbers in the measurement site names indicate the relative measurement position regarding the entire tibia length from distal to proximal. Presented are studies with at least three out of four bone sites. Black points are the mean value of each measurement site. Linear regression analysis showed significant associations across the different studies except for BBR, MEP, and Planhab. ^^ denotes significant relationship with *p* < 0.05; * denotes significant relationship with *p* < 0.01; ^#^ denotes significant relationship with *p* < 0.001.

Figure 6 shows the relationship between ENDO and BL for all studies that provided BMC data for three or more bone sites. Linear regression analysis showed significant associations between ENDO and BL across the different studies (Supplementary Table 3) except for BBR (*p* = 0.93) and MEP (*p* = 0.07). However, these associations disappeared completely, when percent bone losses were plotted against the ratio of endocortical circumference to BMC, a maker of surface-to-volume ratio (Figure 7 and Supplementary Table 4).

Analyses of BMC (mg/mm) of all bone sites with LMM showed that the variability among subjects (SD 47.1 mg/mm) was greater than the variability among studies (SD 4.9 mg/mm), and also greater than residuals (SD 37.7 mg/mm). The residual variability in BMC differed with measurement site: it was about 2.6 times greater for TIBIA_98 than for TIBIA_04, but lower for TIBIA_38 (by the factor 0.5) and TIBIA_66 (by the factor 0.07). For muscle (CSA in mm^2^), the variability among studies (SD 3.0 mm^2^) and subjects (8.9 mm^2^) were similar. Measurement site and study day were significantly associated with BMC and there was no association of ENDO or bed rest duration and BMC. Similar results were observed for CSA (Supplementary Tables 5, 6).

## Discussion

This paper has separated within-subject variation, between-subject variation and measurement uncertainty in a set of control groups from previously bed rest studies, applying a statistical framework for quantifying these aspects. As previously published, bone loss and muscle wasting were generally observed across all studies, underlining the view of Hargens and Vico (2016). The main result of the present paper is that measurement uncertainty of the pQCT was small, and that it cannot explain the large variation of the adaptations after bed rest. In addition, results also demonstrate prominent within-subject variation for bone losses, but not for muscle wasting. Moreover, although some differences were found between studies, the general outcome was relatively homogeneous across studies.

### Measurement Uncertainty

Over the almost two decades during which these studies were performed, we generally note a trend for an improvement in measurement precision, e.g., the difference from 2001/2002 during LTBR till 2015/2016 during RSL. The phenomenon is explained by the steady methodological improvements, especially in the scout view procedures. For example, during LTBR TIBIA_66 positioning was performed manually without scout views, due to the short z-range of the specific XCT2000 device used in that study, which did not allow using distal scout views. Moreover, a proximal scout view was not available at that time. A proximal scout view had become available in the Valdoltra study, albeit in the frontal plane only (Table 2 and Figure 1). These frontal plane knee scout views are often difficult to interpret and definition of a landmark on either of the two knee condyles or on the tibia plateau is inherently difficult. Therefore, introduction of sagittal scout viewing in the RSL study constituted further improvements as such sagittal scouts are much easier to interpret than frontal scout view.

In general, the measurement uncertainty was smaller for bone than for muscle sites. The observation may be explained by the fact that segmentation of muscle cross sections is somewhat more difficult than segmentation of bones. Moreover, orthostasis affects muscle volume. Although all leg muscle measurements were obtained after at least 60 min in the supine position, it is possible that these supine periods prior to pQCT sessions varied across studies, as well as prescription of fluid intake. As fluid redistribution is unlikely to affect BMC, inhomogeneity of fluid redistribution could have contributed to relative large measurement uncertainty for muscle sites. Possibly, different pQCT devices (Table 2) (LTBR: XCT2000 with short z-range; after LTBR XCT-devices with long z-range) are associated with different measurement error, but these are small (0.37% for XCT3000).

Finally, operator experience and skills also have a strong bearing on measurement precision when using pQCT. Therefore, it is recommendable to assess the measurement uncertainty in all bed rest studies, e.g., by performing two baseline measurements. The approach serves to differentiate measurement uncertainty from biological variation between subjects (Swinton et al., 2018) and also provides better estimates of baseline values, thus, increasing the statistical power of the experiment.

### Between-Subject Variation

In general, the responses toward bed rest were homogenous across studies (Figure 2). Turning to between-subject variation, Figure 3 and Table 4 clearly demonstrate that it exists, both for bone loss as well as for muscle wasting, and that between-subject variation was greater for muscle than for bone than for muscle measures. In Figure 3, measurement uncertainty values were remarkably small for TIBIA_04, TIBIA_38 and TIBIA_66, and substantially larger for TIBIA_98 and the muscle sites. Regardless of the confidence interval width, the majority (59.1%) of the individual changes exceeded the interval. Notably, some individual participants showed positive values. The finding implies gains in CSA or BMC in the face of bed rest immobilization. However, such paradoxical gains were observed in the Planhab study only. The Planhab study involved only 21-day of bed rest, and average losses were therefore smaller than in studies with longer bed rest phases. In addition, there was only one baseline measurement in the Planhab study, which led to a less reliable baseline estimate and consequently also to a compromised reliability of the percent change. We therefore speculate that gains in bone mass and muscle CSA measurements may have been produced by a combination of small true changes in the study groups and limited reliability of individual percent changes. However, given the substantial between-subject variation observed in this study, by Scott et al. (2021) and the repeated observation of responders and non-responders to training interventions (McPhee et al., 2010; Mann et al., 2014; Hecksteden et al., 2015; Ahtiainen et al., 2016; Ross et al., 2019), blunted or even paradoxical responses to bed rest cannot be ruled out. We suggest that future bed rest studies should make further attempts at improving and unifying standard operating procedures for pQCT. In particular, two separate baseline measurements should be included whenever possible.

Figure 4 shows that the adjusted between-subject deviation (i.e., uncertainty of individual response relative to the averaged change per week for each study group) was greater for bone than for muscle. The observation is also confirmed by statistical analysis with linear mixed model, which has shown smaller adjusted between-subject deviation for muscle than for bone sites. Due to the fact that this manuscript referred to the data sets of the control groups only, it was impossible to use the already established approach of Hopkins (2015) and Atkinson and Batterham (2015), who compared the standard deviation of control and intervention group.

### Within-Subject Variation

In addition to between-subject variation, the present study has also explored variation within subjects. A previous publication from the LTBR study reported significant correlation of bone losses at Tibia 66% with the other bone sites, but no such correlation among these other sites (Rittweger et al., 2005). In the present analysis of a much larger data base, these findings are replicated in that TIBIA_66 losses were correlated with losses at all other bone sites, even with the loss of compact bone tissue at TIBIA_04, but not at TIBIA_98. Yet, the present work did find correlations among the other bone sites, except between TIBIA_38 and TIBIA_98 (Figure 5 and Table 5). These significant correlations are consistent with individual traits in the bed rest responses of bone and muscle. However, all inter-bone site correlations (r between 0.34 and 0.52) were substantially weaker than the inter-muscle site correlation (*r* = 0.90). Looking at figures in Table 5, one might recognize a pattern of stronger correlations among diaphyseal sites (TIBIA_38 and TIBIA_66, *r* = 0.52), and of weaker correlations between epiphyseal and diaphyseal sites (*r*-values ranging between 0.29 and 0.51). Additionally, differentiating into compact and trabecular bone tissue at the epiphyseal bone sites showed that there were significant correlations between TIBIA_04_Cort and the diaphyseal sites, but no correlation to TIBIA_98_Cort, which could be explained by the fact that the compact bone tissue is often not thicker than the trabeculi at TIBIA_98. The comparison of the trabecular bone loss of the epiphyseal sites did not show any correlation, too. Overall, there seems to be little variability in the bed rest response within an individual’s calf musculature, while the tibia’s response exhibits substantial within-subject variation, even after dividing the epiphyseal measurement sites into compact and trabecular bone tissue.

Finally, it was observed that there were significant correlations between bone losses and muscle wasting, which are large for TIBIA_98 vs. either muscle sites (Table 5), but only moderate at best for the diaphyseal bone sites and for TIBIA_04. Bones adapt their structure to their mechanical environment (Frost, 1987; Rubin and Lanyon, 1987), and the greatest forces that bones are exposed to originate from regional muscle contractions (Rittweger, 2007). Consequently, bone strength measures typically depict large correlations with measures of muscle strength (Schiessl et al., 1998; Rittweger et al., 2000). Accordingly, previous studies had hypothesized to find correlations between individual muscle wasting and bone losses. However, such correlations never substantiated (Rittweger et al., 2005, 2010), at least at the diaphyseal and distal epiphyseal tibia sites and it had been proposed that bed rest is permissive, rather than inductive of bone loss (Rittweger et al., 2005). Large and highly significant correlations for the proximal epiphysis, which were observed in the present paper, are therefore unexpected. The question in how far the distal and proximal epiphysis differ deserves further study beyond the differences presented in this manuscript regarding adaptation of compact and trabecular bone tissue. However, the present study has much greater sample size than the aforementioned papers. Finding of a joint muscle-bone response also resonates with a recent report that habitual physical activity predicts space-flight induced bone losses (Gabel et al., 2021).

### Origins of Variability

Muscles and bones fulfill mechanical roles for our organism and bed rest is foremost a model for the withdrawal of mechanical challenges. This withdrawal encompasses not only any habitual locomotor activities, but also participation in exercise and sports and it is also associated with metabolic derailments such as insulin resistance (Biensø et al., 2012). Given that there likely was substantial between-subject variation in exercise participation prior to participating in the bed rest studies, one could hypothesize that reductions in mechanical challenges varied between subjects, and that this variable reduction constitutes one origin of variability in bed rest response. Bed rest as well as spaceflight data support the idea (Rittweger et al., 2005; Gabel et al., 2021). Similarly, pre-study dietary habits may have varied between subjects, while the diet that is typically provided in ESA or NASA-funded bed rest studies is highly standardized. In many cases that diet will considerably deviate from the habitual intake patterns with expected effects on metabolism and adaptive processes. In addition, the well-established view of individual responsiveness to exercise interventions (McPhee et al., 2010; Mann et al., 2014; Hecksteden et al., 2015; Ahtiainen et al., 2016; Ross et al., 2019) also needs to be considered. This variable responsiveness may result from genetic and epigenetic pre-dispositions, which has been demonstrated, e.g., for the ACE ii/dd polymorphism (Montgomery et al., 1999; Valdivieso et al., 2017). Quite as much as in responses to increased mechanical and metabolic challenges, as in exercise training, genetic predispositions could also modulate the response to bed rest.

Finally, sizable variation in bone losses was observed between different tibia sites and bone tissues, but not between muscle sites. As a conclusion, changes in lifestyle like exercising and nutrition as well as genetic predisposition may largely explain variation in muscle wasting. But obviously, there may be additional factors causing the different inter-site bone losses. Possibly, the differences between muscle and bone variation could be explained by their participation in metabolic processes. For bone, the metabolic involvement is primarily in calcium and phosphate. Of the 1,000 g calcium in the human body, only 1 g is located outside bone. Moreover, rapid calcium transients cause electrophysiological disruptions that are potentially lethal. Accordingly, serum calcium levels have to be kept within bands that are extremely narrow when considering that the bone reservoir is 1,000 times larger than the extra-osseous pool. Moreover, bone surfaces are covered by bone lining cells, which constitute a relatively small amount of biomass in relation to the huge bone mass, and whose function it is to separate the ionic milieu in bone from the other fluid spaces (Rubinacci et al., 2002; Marenzana et al., 2005). High calcium-phosphate levels in the body fluids are known to foster extra-osseous calcifications, and, sub-clinical renal calculus formation can occur in spaceflight and bed rest alike, highlighting possible limitations in phospho-calcic excretion capability (Watanabe et al., 2004). Naturally, between-subject variation can be pertinent to that capability as well. This all results in the adaptive responses in bone being relatively slow, potentially with a variable degree of individual traits. Hence, extra-osseous factors involved in the handling end excretion of calcium could constitute another important source of between-subject variation in spaceflight- and immobilized bone losses.

Another important peculiarity of bone is the cellular mechanism by which it is degraded. When active, multi-nucleated osteoclasts resorb bone in a specific space, contrasting with skeletal muscle, where protein degradation likely occurs more uniformly in all cells. Moreover, different bone turnover rates strongly differ between tissue compartments as shown for the iliac crest by Balena et al. (1992), which is not direct transferable to the tibia, but could be a clue for osseous within-subject variation. These compartment-specific differences in the bone’s remodeling activity are likely the origin of the correlation between endocortical perimeter and BMC losses in Figure 6, thus confirming a previous finding. However, when using the ratio of endocortical perimeter to BMC as a marker of surface-to-volume ratio, all correlations with percent bone losses disappeared, indicating that bone “geometry” indicators do not predict individual losses (Figure 7). In addition, the linear mixed model showed that there was no association between endocortical circumference and BMC (Supplementary Table 5).

### Preventing Worst Case Scenarios

Bed rest studies have so far focused on decrements in bone mass, muscle size etc., that were averaged within groups, and that were compared between control and countermeasure groups. This approach is straightforward to treat population means, but it could be problematic for single individuals. Provided the average bone loss in a long-term space mission at a given site is 1% per month and the between-subject SD of that loss is also 1% per month, the expected average loss is 12% during a 12-month mission. In addition, Gaussian distribution would predict that the largest bone loss in a 6-person crew will amount to 12% + 12% ⋅ 0.967 = 23.6%, with 0.967 being the upper tail quantile for 1/6 of the normal distribution. Therefore, when the aim is to safeguard the strongest responder to microgravity exposure of the crew members, a better understanding of between-subject variation becomes as important as averaged effectiveness of countermeasures.

### Consequences of Variability

These above findings have important implications for the design and interpretation of bed rest studies. With regards to sample size estimation, if the scientists are interested in mean effects, one way to enhance study power is to increase the number of participants. Alternatively, one might try to diminish the influence of individual variations in study endpoints by better controlling for habitual physical activity and dietary habits. These covariates are highly controlled during, but not prior to the bed rest studies. However, subject recruitment for bed rest studies is already quite a challenge and expanding the list of inclusion and exclusion criteria would certainly hamper the feasibility of such studies. Moreover, even if it was possible to fully homogenize the response to the bed rest between subjects, the same homogenization would probably not be feasible for space missions. Therefore, it might be best to monitor, rather than to control putative pre-bed rest covariates in the future. At least, the habitual daily diet and physical activity need to be assessed by a detailed questionnaire. Definitely, these aspects should be transferred to future Astronaut recruitment, increased by analysis of the genetic predisposition and hormone analysis to make full use of the current available possibilities of individual response predictions. Especially the manned missions to Moon and Mars, which lasts clearly longer than the current missions and will have a greater demand on physical health, make an intense analysis of the predisposition regarding greater individual variations in bone loss and muscle wasting imperative.

### Limitations

The assessment of measurement uncertainty was limited as only three out of eight studies provided two baseline measurements and the evaluated measurement sites differed among studies. To overcome this, the authors summarized results using the most similar condition. Enhanced estimation of measurement uncertainty could be achieved by increasing the baseline measurements of the same subjects on different days without any intervention. The analyses focused only on individual data of the control groups, thus the results of this paper are just valid for participants without any additional intervention besides bed rest. A further investigation of the intervention groups is needed. The intervention groups were excluded, because the aim of the paper was to do a first exploration of the relationship of measurement uncertainty, between-subject variation and within-subject variation, thus, additional intervention next to bed rest would complicate this approach. It needs to be mentioned that some of the included data sets are from cross-over design studies (Planhab, MEP, NUC), where participants underwent additional intervention, but there were well-dosed wash-out phases between interventions. The present evidence base comprises only two lower leg muscle sites and four tibia bone sites. However, obtaining data from more diverging bone and muscle sites would demand substantially greater subject time budgets, and also probably different technology such as full-size computed tomography or magnetic resonance imaging. By contrast, the strength of our data base is that it is used one single technology. Therefore, we trust that the principles of within-subject and between-subject variation in bone and muscle responses to bed rest, which have been firstly laid out here, may also apply to other anatomical sites. Yet, past studies have already shown that there is no bone loss in the upper extremity after bed rest (Hargens and Vico, 2016). Lastly, there may be additional factors affecting individual response that have not been captured.

## Conclusion

Variation in muscle and bone responses to bed rest primarily results from between-subject and within-subject variation rather than measurement uncertainty. Nevertheless, measurement uncertainty should be considered in each data analysis, regardless of variation. It was observed that between-subject variation and within-subject variation were both lower for muscle than for bone sites. Training status, diet, and genetic predisposition may have contributed to the variation. The substantial variation in bone and muscle responses to deconditioning, be it in bed rest or during space missions, provides an impetus for a more individualized approach to countermeasure prescription.

## Data Availability Statement

The original contributions presented in the study are included in the article/Supplementary Material, further inquiries can be directed to the corresponding author/s.

## Ethics Statement

The studies involving human participants were reviewed and approved by the local ethics committees. The patients/participants provided their written informed consent to participate in this study.

## Author Contributions

JB and JR created the idea of the manuscript. JB, M-TS, UM, and JR worked on the statistical approach. JR and UM provided the data of the measurements. JB analyzed the data and wrote the manuscript. JB, JR, M-TS, UM, and JJ edited and revised the manuscript. All authors contributed to the article and approved the submitted version.

## Conflict of Interest

The authors declare that the research was conducted in the absence of any commercial or financial relationships that could be construed as a potential conflict of interest.

## Publisher’s Note

All claims expressed in this article are solely those of the authors and do not necessarily represent those of their affiliated organizations, or those of the publisher, the editors and the reviewers. Any product that may be evaluated in this article, or claim that may be made by its manufacturer, is not guaranteed or endorsed by the publisher.

## Footnotes

1
www.r-project.org
