# Corrigendum: Between-Subject and Within-Subject Variation of Muscle Atrophy and Bone Loss in Response to Experimental Bed Rest

**DOI:** 10.3389/fphys.2022.913252

**Published:** 2022-05-12

**Authors:** Jonas Böcker, Marie-Therese Schmitz, Uwe Mittag, Jens Jordan, Jörn Rittweger

**Affiliations:** ^1^ Department of Muscle and Bone Metabolism, German Aerospace Center, Institute of Aerospace Medicine, Cologne, Germany; ^2^ Institute of Medical Biometry, Informatics and Epidemiology (IMBIE), University Hospital Bonn, Bonn, Germany; ^3^ Chair of Aerospace Medicine, University of Cologne, Cologne, Germany; ^4^ German Aerospace Center, Head of Institute of Aerospace Medicine, Cologne, Germany; ^5^ Department of Pediatrics and Adolescent Medicine, University Hospital of Cologne, Cologne, Germany

**Keywords:** between-subject variation, within-subject variation, measurement uncertainty, bed rest, muscle atrophy, bone loss

In the original article, there was a mistake in Figure 3 as published. The confidence intervals were partly not correctly defined. The corrected Figure 3 appears below.

**FIGURE 3:**
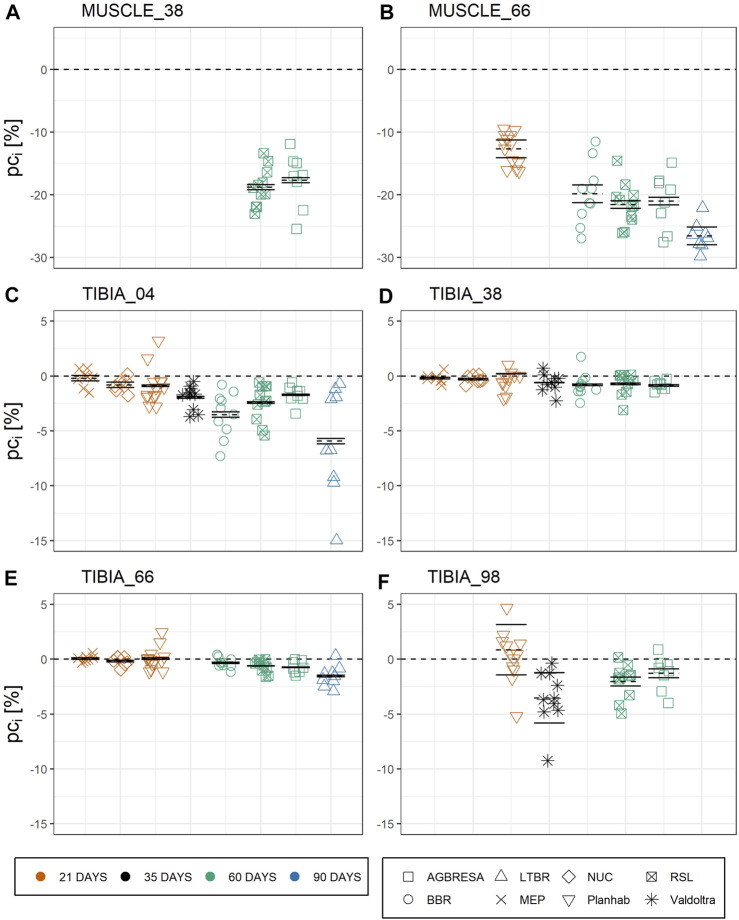
Chart of the individual percent change (*pc*_*i*_) by measurement sites with **(A)** CSA at MUSCLE_38, **(B)** CSA at MUSCLE_66, **(C)** BMC at TIBIA_04, **(D)** BMC at TIBIA_38, **(E)** BMC at TIBIA_66, and **(F)** BMC at TIBIA_98, where the numbers indicate the relative measurement position regarding the entire tibia length from distal to proximal. The color indicates the bed rest duration and the shape represents the study. Each chart is separated by the studies, who performed measurements at the measurement site. Mean of the pc as dashed line, upper and lower limit of the 95%-confidence interval based on measurement uncertainty *U*_*Meas*_ as solid lines. The vast majority of *pc*_*i*_ exceeds the confidence interval indicating significant and substantial between-subject variation.

Furthermore, the confidence intervals of the between-subject variation were partly not correctly defined. Because of that, there is a change in one number that occurs three times in the text.

A correction has been made to the **Abstract**. The corrected section appears below:

“To improve quantification of individual responses to bed rest interventions, we analyzed peripheral quantitative computer tomography (pQCT) datasets of the lower leg of 76 participants, who took part in eight different bed rest studies. A newly developed statistical approach differentiated measurement uncertainty *U*_*Meas*_ from between-subject variation (BSV) and within-subject variation (WSV). The results showed that *U*_*Meas*_ decreased 59.3%–80% over the two decades of bed rest studies (*p* < 0.01), and that it was higher for muscles than for bones. The reduction of *U*_*Meas*_ could be explained by improved measurement procedures as well as a higher standardization. The vast majority (82.6%) of the individual responses *pc*_*i*_ exceeded the 95% confidence interval defined by *U*_*Meas*_, indicating significant and substantial BSV, which was greater for bones than for muscles, especially at the epiphyseal measurement sites. Non-significant to small positive inter-site correlations between bone sites, but very large positive inter-site correlation between muscle sites suggests that substantial WSV exists in the tibia bone, but much less so in the calf musculature. Furthermore, endocortical circumference, an indicator of the individual’s bone geometry could partly explain WSV and BSV. These results demonstrate the existence of substantial BSV bone, and that it is partly driven by WSV, and likely also by physical activity and dietary habits prior to bed rest. In addition, genetic and epigenetic variation could potentially explain BSV, but not WSV. As to the latter, differences of bone characteristics and the bone resorption process could offer an explanation for its existence. The study has also demonstrated the importance of duplicate baseline measurements. Finally, we provide here a rationale for worst case scenarios with partly effective countermeasures in long-term space missions.”

A correction has been made to **Results**, paragraph 2. The corrected paragraph appears below:

“For computation of the measurement uncertainty *U*_*Meas*_, we used the results of the studies with two baseline measurements, meaning LTBR, RSL, and Valdoltra, respectively. ANOVA indicated a significant difference of *U*_*Meas*_ between RSL and LTBR at TIBIA_66 (*p* = 0.005) (**Table 3**). As can be seen from Figure 3, the vast majority (82.6%) of the observed individual percent change *pc*_*i*_ exceeds the confidence intervals, indicating significant and substantial BSV. By subtracting the calculated *U*_*Meas*_ from *U*_*Obs*_, *U*_*IR*_ was calculated (**Table 4**).”

A correction has been made to **Discussion, Between-Subject Variation**, paragraph 1. The corrected paragraph appears below:

“In general, the responses toward bed rest were homogenous across studies (**Figure 2**). Turning to between-subject variation, Figure 3 and **Table 4** clearly demonstrate that it exists, both for bone loss as well as for muscle wasting, and that between-subject variation was greater for muscle than for bone measures. In Figure 3, measurement uncertainty values were remarkably small for TIBIA_04, TIBIA_38 and TIBIA_66, and substantially larger for TIBIA_98 and the muscle sites. Regardless of the confidence interval width, the vast majority (82.6%) of the individual changes exceeded the interval. Notably, some individual participants showed positive values. The finding implies gains in CSA or BMC in the face of bed rest immobilization. However, such paradoxical gains were observed in the Planhab study only. The Planhab study involved only 21-day of bed rest, and average losses were therefore smaller than in studies with longer bed rest phases. In addition, there was only one baseline measurement in the Planhab study, which led to a less reliable baseline estimate and consequently also to a compromised reliability of the percent change. We therefore speculate that gains in bone mass and muscle CSA measurements may have been produced by a combination of small true changes in the study groups and limited reliability of individual percent changes. However, given the substantial between-subject variation observed in this study, by Scott et al. (2021) and the repeated observation of responders and non-responders to training interventions (McPhee et al., 2010; Mann et al., 2014; Hecksteden et al., 2015; Ahtiainen et al., 2016; Ross et al., 2019), blunted or even paradoxical responses to bed rest cannot be ruled out. We suggest that future bed rest studies should make further attempts at improving and unifying standard operating procedures for pQCT. In particular, two separate baseline measurements should be included whenever possible.”

The authors apologize for this error and state that this does not change the scientific conclusions of the article in any way. The original article has been updated.

