# Corrigendum: Between-subject and within-subject variation of muscle atrophy and bone loss in response to experimental bed rest

**DOI:** 10.3389/fphys.2024.1528096

**Published:** 2025-01-03

**Authors:** Jonas Böcker, Marie-Therese Schmitz, Uwe Mittag, Jens Jordan, Jörn Rittweger

**Affiliations:** ^1^ Department of Muscle and Bone Metabolism, German Aerospace Center, Institute of Aerospace Medicine, Cologne, Germany; ^2^ Institute of Medical Biometry, Informatics and Epidemiology (IMBIE), University Hospital Bonn, Bonn, Germany; ^3^ Chair of Aerospace Medicine, University of Cologne, Cologne, Germany; ^4^ German Aerospace Center, Head of Institute of Aerospace Medicine, Cologne, Germany; ^5^ Department of Pediatrics and Adolescent Medicine, University Hospital of Cologne, Cologne, Germany

**Keywords:** between-subject variation, within-subject variation, measurement uncertainty, bed rest, muscle atrophy, bone loss

In the published article, there were some errors.

A correction has been made to the **Abstract**. The sentences previously stated:

The vast majority (82.6%) of the individual responses *pc*_*i*_ exceeded the 95% confidence interval defined by *U*_*Meas*_, indicating significant and substantial BSV, which was greater for bones than for muscles, especially at the epiphyseal measurement sites … These results demonstrate the existence of substantial BSV bone, and that it is partly driven by WSV, and likely also by physical activity and dietary habits prior to bed rest. In addition, genetic and epigenetic variation could potentially explain BSV, but not WSV.

The corrected sentences appear below:

The majority (59.1%) of the individual responses *pc*_*i*_ exceeded the 95% confidence interval defined by *U*_*Meas*_, indicating significant and substantial BSV, which was greater for bones than for muscles, especially at the diaphyseal measurement sites…. These results demonstrate the existence of substantial bone BSV and that it is partly driven by WSV and likely also by physical activity and dietary habits prior to bed rest.

A correction has been made to **Results, paragraph 2**. The sentences previously stated:

As can be seen from Figure 3, the vast majority (82.6%) of the observed individual percent change *pc*_*i*_ exceeds the confidence intervals, indicating significant and substantial BSV. By subtracting the calculated *U*_*Meas*_ from *U*_*Obs*_, *U*_*IR*_ was calculated (**Table 4**).

**FIGURE 3:**
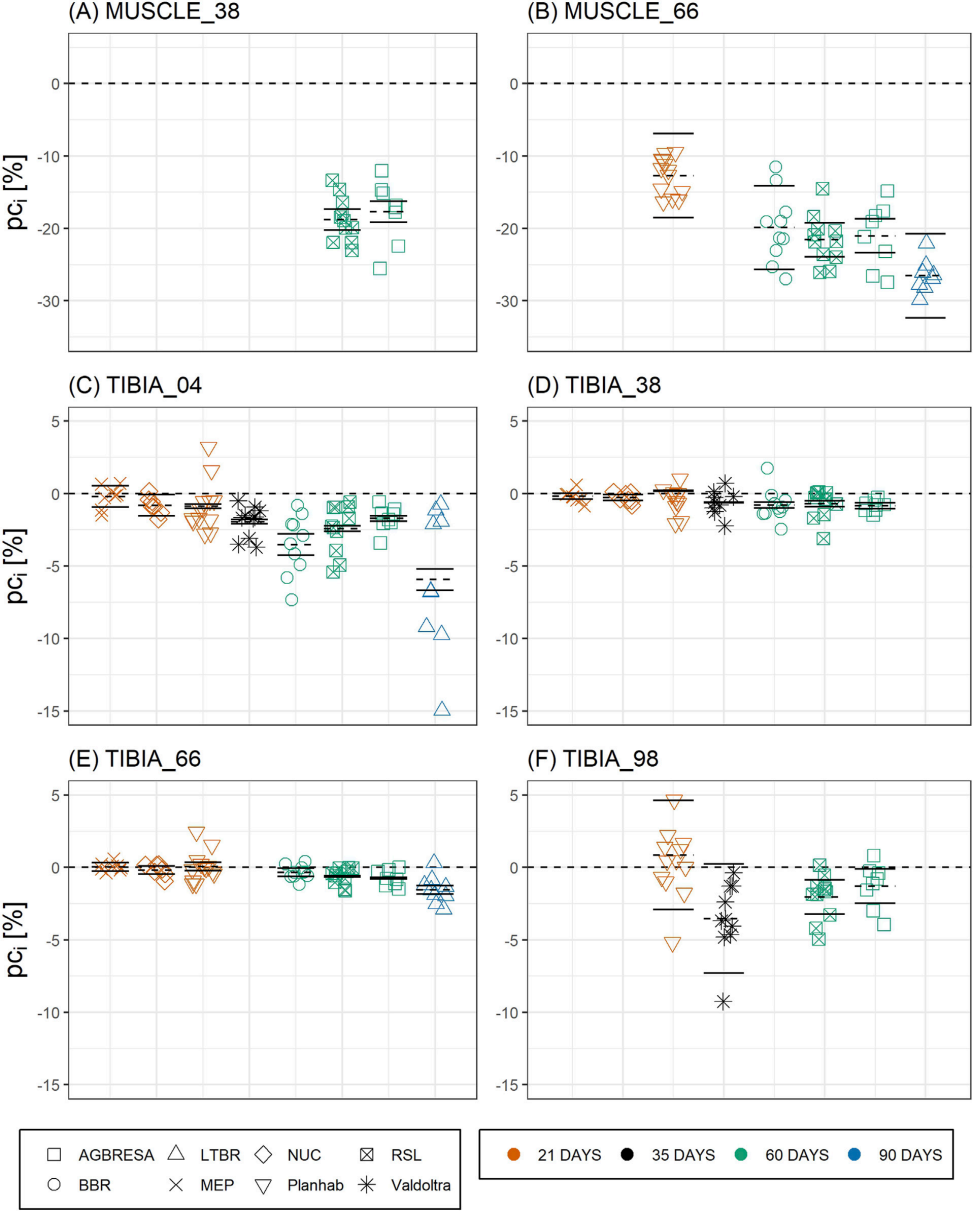
Chart of the individual percent change (*pc*_*i*_) by measurement sites with **(A)** CSA at MUSCLE_38, **(B)** CSA at MUSCLE_66, **(C)** BMC at TIBIA_04, **(D)** BMC at TIBIA_38, **(E)** BMC at TIBIA_66, and **(F)** BMC at TIBIA_98, where the numbers indicate the relative measurement position regarding the entire tibia length from distal to proximal. The color indicates the bed rest duration and the shape represents the study. Each chart is separated by the studies that performed measurements at the measurement site. Mean of the pc as dashed line, upper and lower limit of the 95%-confidence interval based on measurement uncertainty *U*_*Meas*_ as solid lines. Most *pc*_*i*_ exceed the confidence interval, indicating significant between-subject variation.

The corrected sentences appear below:

As can be seen from Figure 3, the majority (59.1%) of the observed individual percent change *pc*_*i*_ exceeds the confidence intervals, indicating significant and substantial BSV. However, the BSV was greater for bone (65.9%) than for muscle (34.8%), and the BSV was greater for the diaphysis (71.0%) than for the epiphyseal measurement sites (60.2%).

A correction has been made to **Discussion**, *subsection Between-Subject Variation*. The sentences previously stated:

Turning to between-subject variation, Figure 3 and **Table 4** clearly demonstrate that it exists, both for bone loss as well as for muscle wasting, and that between-subject variation was greater for muscle than for bone measures. In Figure 3, measurement uncertainty values were remarkably small for TIBIA_04, TIBIA_38 and TIBIA_66, and substantially larger for TIBIA_98 and the muscle sites. Regardless of the confidence interval width, the vast majority (82.6%) of the individual changes exceeded the interval.

The corrected sentences appear below:

Turning to between-subject variation, Figure 3 and **Table 4** clearly demonstrate that it exists, both for bone loss as well as for muscle wasting, and that between-subject variation was greater for bone than for muscle measures. In Figure 3, measurement uncertainty values were remarkably small for TIBIA_04, TIBIA_38 and TIBIA_66, and substantially larger for TIBIA_98 and the muscle sites. Regardless of the confidence interval width, the majority (59.1%) of the individual changes exceeded the interval.

A correction has been made to **Figure 3** caption.

FIGURE 3 Chart of the individual percent change (*pc*_*i*_) by measurement sites with **(A)** CSA at MUSCLE_38, **(B)** CSA at MUSCLE_66, **(C)** BMC at TIBIA_04, **(D)** BMC at TIBIA_38, **(E)** BMC at TIBIA_66, and **(F)** BMC at TIBIA_98, where the numbers indicate the relative measurement position regarding the entire tibia length from distal to proximal. The color indicates the bed rest duration and the shape represents the study. Each chart is separated by the studies, who performed measurements at the measurement site. Mean of the pc as dashed line, upper and lower limit of the 95%-confidence interval based on measurement uncertainty *U*_*Meas*_ as solid lines. The vast majority of *pc*_*i*_ exceeds the confidence interval indicating significant and substantial between-subject variation.

The corrected caption appears below.

FIGURE 3 Chart of the individual percent change (*pc*_*i*_) by measurement sites with **(A)** CSA at MUSCLE_38, **(B)** CSA at MUSCLE_66, **(C)** BMC at TIBIA_04, **(D)** BMC at TIBIA_38, **(E)** BMC at TIBIA_66, and **(F)** BMC at TIBIA_98, where the numbers indicate the relative measurement position regarding the entire tibia length from distal to proximal. The color indicates the bed rest duration and the shape represents the study. Each chart is separated by the studies, who performed measurements at the measurement site. Mean of the pc as dashed line, upper and lower limit of the 95%-confidence interval based on measurement uncertainty *U*_*Meas*_ as solid lines. The vast majority of *pc*_*i*_ exceeds the confidence interval indicating significant and substantial between-subject variation.

The authors apologize for this error and state that this does not change the scientific conclusions of the article in any way.

