# Supplementary material for: Between-Subject and Within-Subject Variation of Muscle Atrophy and Bone Loss in Response to Experimental Bed Rest
Source: Front Physiol. 2022 Feb 22;12:743876. doi: 10.3389/fphys.2021.743876 (PMC8902302; doi:10.3389/fphys.2021.743876)
Supplement: Supplementary file 1 [file Table_1.pdf]

Supplementary Material

Table 1: Percent change pc_k [%] of adaptation of CSA (MUSCLE_38 and MUSCLE_66) and BMC (TIBIA_04, TIBIA_38, TIBIA_66, TIBIA_98) as mean ± standard deviation after bed rest by body sites and studies. In brackets the minimum and maximum. p-values indicates whether there was a significant difference between baseline and after bed rest.

k	Study	MUSCLE_38	MUSCLE_66	TIBIA_04	TIBIA_38	TIBIA_66	TIBIA_98
1	AGBRESA	-17.67 ± 4.34<br>(-25.42; -12.01)<br>p = 0.003	-21.01 ± 4.47<br>(-27.53; -14.75)<br>p = 0.01	-1.71 ± 0.85<br>(-3.43; -0.57)<br>p = 0.83	-0.85 ± 0.41<br>(-1.50; -0.25)<br>p = 0.90	-0.74 ± 0.55<br>(-1.53; -0.03)<br>p = 0.91	-1.29 ± 1.55<br>(-4.02; 0.85)<br>p = 0.89
2	BBR	-	-19.87 ± 4.86<br>(-26.95; -11.51)<br>p < 0.001	-3.52 ± 2.07<br>(-7.36; -0.82)<br>p = 0.55	-0.80 ± 1.09<br>(-2.45; 1.76)<br>p = 0.81	-0.33 ± 0.46<br>(-1.16; 0.40)<br>p = 0.91	-
3	LTBR	-	-26.54 ± 2.32<br>(-29.76; -21.99)<br>p < 0.001	-5.93 ± 4.86<br>(-14.98; -0.72)<br>p = 0.39	-	-1.54 ± 0.94<br>(-2.89; 0.32)<br>p = 0.72	-
4	MEP	-	-	-0.20 ± 0.78<br>(-1.49; 0.72)<br>p = 0.98	-0.19 ± 0.42<br>(-0.85; 0.62)<br>p = 0.96	0.05 ± 0.29<br>(-0.35; 0.57)<br>p = 0.99	-
5	NUC	-	-	-5.98 ± 8.10<br>(-1.76; 0.18)<br>p = 0.90	-0.28 ± 0.35<br>(-0.83; 0.13)<br>p = 0.96	-0.18 ± 0.43<br>(-0.95; 0.21)<br>p = 0.97	-
6	Planhab	-	-12.70 ± 2.52<br>(-16.27; -9.45)<br>p = 0.07	-0.88 ± 1.68<br>(-2.80; 3.22)<br>p = 0.88	0.20 ± 2.42<br>(-2.06; 7.39)<br>p = 0.97	0.05 ± 1.02<br>(-1.14; 2.46)<br>p = 0.99	0.85 ± 3.03<br>(-5.18; 7.41)<br>p = 0.90
7	RSL	-18.78 ± 3.06<br>(-21.84; -15.72)<br>p = 0.002	-21.58 ± 3.36<br>(-26.08; -14.54)<br>p < 0.001	-2.42 ± 1.68<br>(-5.42; -0.59)<br>p = 0.60	-0.71 ± 1.00<br>(-3.11; 0.12)<br>p = 0.86	-0.61 ± 0.57<br>(-1.61; -0.03)<br>p = 0.90	-2.05 ± 1.52<br>(-4.96; 0.15)<br>p = 0.71
8	Valdoltra	-	-	-1.94 ± 1.11<br>(-3.71; -0.51)<br>p = 0.75	-0.61 ± 0.86<br>(-2.24; 0.73)<br>p = 0.91	-	-3.54 ± 2.52<br>(-9.25; -0.36)<br>p = 0.57
