# Supplementary material for: Between-Subject and Within-Subject Variation of Muscle Atrophy and Bone Loss in Response to Experimental Bed Rest
Source: Front Physiol. 2022 Feb 22;12:743876. doi: 10.3389/fphys.2021.743876 (PMC8902302; doi:10.3389/fphys.2021.743876)
Supplement: Supplementary file 2 [file Table_2.pdf]

# *Supplementary Material*

Table 2: Results of ANOVA and Tukey HSD for significant difference of $U_{Obs}$ and $U_{IR}$ between measurement sites. p-values are shown with the result for $U_{Obs}$ at the top and $U_{IR}$ at the bottom. Significant differences are marked in grey.

| Measurement Site | MUSCLE_38 | MUSCLE_66 | TIBIA_04 | TIBIA_38 | TIBIA_66 | TIBIA_98 |
|---|---|---|---|---|---|---|
| MUSCLE_38 | - | 0.99<br>0.99 | 0.20<br>0.25 | <0.05<br>0.07 | 0.03<br>0.04 | 0.36<br>0.30 |
| MUSCLE_66 | 0.99<br>0.99 | - | <0.05<br>0.20 | 0.005<br>0.03 | 0.003<br>0.02 | 0.19<br>0.31 |
| TIBIA_04 | 0.20<br>0.25 | <0.05<br>0.20 | - | 0.87<br>0.89 | 0.71<br>0.73 | 0.99<br>0.99 |
| TIBIA_38 | <0.05<br>0.07 | 0.005<br>0.03 | 0.87<br>0.89 | - | 0.99<br>0.99 | 0.86<br>0.97 |
| TIBIA_66 | 0.03<br>0.04 | 0.003<br>0.02 | 0.71<br>0.73 | 0.99<br>0.99 | - | 0.72<br>0.89 |
| TIBIA_98 | 0.36<br>0.30 | 0.19<br>0.31 | 0.99<br>0.99 | 0.86<br>0.97 | 0.72<br>0.89 | - |
