# Supplementary material for: Between-Subject and Within-Subject Variation of Muscle Atrophy and Bone Loss in Response to Experimental Bed Rest
Source: Front Physiol. 2022 Feb 22;12:743876. doi: 10.3389/fphys.2021.743876 (PMC8902302; doi:10.3389/fphys.2021.743876)
Supplement: Supplementary file 3 [file Table_3.pdf]

Supplementary Material

Table 3: Results of linear regression analysis for ENDO and BL with r^2, r, p-values, beta and standard error (Std Error) by each study and all combined studies.

k	Study	r^2	r	p-value	Beta	Std Error
1	AGBRESA	0.29	0.54	0.001	0.05	0.01
2	BBR	< 0.001	0.02	0.93	0.01	0.10
4	MEP	0.14	0.37	0.07	0.01	0.01
5	NUC	0.32	0.56	0.008	0.03	0.01
6	Planhab	0.22	0.47	< 0.001	0.09	0.02
7	RSL	0.40	0.64	< 0.001	0.09	0.02
8	Valdoltra	0.46	0.68	< 0.001	0.15	0.03
	All studies	0.30	0.55	< 0.001	0.09	0.01
