# Supplementary material for: Between-Subject and Within-Subject Variation of Muscle Atrophy and Bone Loss in Response to Experimental Bed Rest
Source: Front Physiol. 2022 Feb 22;12:743876. doi: 10.3389/fphys.2021.743876 (PMC8902302; doi:10.3389/fphys.2021.743876)
Supplement: Supplementary file 4 [file Table_4.pdf]

Supplementary Material

Table 4: Results of linear regression analysis for ENDO-to-BMC-ratio and pc_i with r^2, r, p-values, beta and standard error of the coefficient by each study and all combined studies.

k	Study	r^2	r	p	Beta	Std Error
1	AGBRESA	0.16	0.40	0.02	-4.74	1.98
2	BBR	0.05	0.22	0.25	-12.34	10.50
4	MEP	0.01	0.73	0.61	-0.63	1.23
5	NUC	0.33	0.58	0.006	-3.37	1.09
6	Planhab	0.01	0.10	0.47	-2.54	3.48
7	RSL	0.35	0.59	< 0.001	-9.71	2.03
8	Valdoltra	0.29	0.54	0.003	-12.74	3.84
	All studies	0.07	0.27	< 0.001	-5.58	1.31
