# Supplementary material for: Between-Subject and Within-Subject Variation of Muscle Atrophy and Bone Loss in Response to Experimental Bed Rest
Source: Front Physiol. 2022 Feb 22;12:743876. doi: 10.3389/fphys.2021.743876 (PMC8902302; doi:10.3389/fphys.2021.743876)
Supplement: Supplementary file 5 [file Table_5.pdf]

Supplementary Material

Table 5: Results of the linear mixed model (LMM) analyzing the effects of several factors on BMC.

Factors	Beta	Std Error	p value
ENDO	0.16	0.10	0.11
Measurement site (ref= TIBIA_04)			<0.001
Measurement Site TIBIA_38	6.10	8.36	
Measurement Site TIBIA_66	50.17	6.36	
Measurement Site TIBIA_98	442.80	15.31	
Study Day post bed rest	-2.19	0.47	<0.001
Bed rest (ref = 60 Days)			0.45
Bed rest 21 Days	-17.87	13.16	
Bed rest 35 Days	-8.12	18.74	
Bed rest 90 Days	-24.57	18.88	
