# Supplementary material for: Between-Subject and Within-Subject Variation of Muscle Atrophy and Bone Loss in Response to Experimental Bed Rest
Source: Front Physiol. 2022 Feb 22;12:743876. doi: 10.3389/fphys.2021.743876 (PMC8902302; doi:10.3389/fphys.2021.743876)
Supplement: Supplementary file 6 [file Table_6.pdf]

Supplementary Material

Table 6: Results of the linear mixed model (LMM) analyzing the effects of several factors on CSA.

Factors	Beta	Std Error	p value
Measurement Site MUSCLE_66	32.29	2.59	< 0.001
Study Day post bed rest	-13.67	0.80	< 0.001
Bed rest (ref = 60 Days)			0.46
Bed rest 21 Days	4.61	4.71	
Bed rest 90 Days	-2.65	5.15	
